# Geriatric syndromes in an urban elderly population in Cameroon: a focus on disability, sarcopenia and cognitive impairment

**DOI:** 10.11604/pamj.2020.37.229.26634

**Published:** 2020-11-11

**Authors:** Marie Josiane Ntsama Essomba, Daniel Atsa, Dimitri Zogo Noah, Marylin Zingui-Ottou, Ginette Paula, Jan René Nkeck, Jean Jacques Noubiap, Gloria Ashuntantang

**Affiliations:** 1 Geriatric Unit, Yaoundé Central Hospital, Yaoundé, Cameroon,; 2 Department of Internal Medicine and Specialties, University of Yaoundé I, Yaoundé, Cameroon,; 3 Yaoundé Emergencies Center, Yaoundé, Cameroon,; 4 Center for Heart Rhythm Disorders, University of Adelaide and Royal Adelaide Hospital, Adelaide, Australia

**Keywords:** Geriatric syndromes, disability, sarcopenia, cognitive impairment, Cameroon

## Abstract

**Introduction:**

geriatric syndromes are multifactorial conditions that are associated with substantial disability, poor quality of life and mortality in the elderly. The patterns of these conditions are poorly described in sub-Saharan Africa. This study aimed to determine the prevalence and correlates of common geriatric syndromes in Cameroon.

**Methods:**

we conducted a cross-sectional study in the geriatrics unit of a university hospital in Cameroon. All people aged ≥55 who attended a health promotion and screening campaign in September 2019 were included. Geriatric syndromes including functional decline, cognitive impairment and sarcopenia were assessed. We also examined sociodemographic characteristics and comorbidities.

**Results:**

overall, 104 participants were enrolled with median age of 65 (IQR: 62.2 - 70.8). About 67% of participants presented at least one geriatric syndrome. Disability in activities of daily living and instrumental activities of daily living were present in 10% and 38% of participants respectively and associated factors were male gender (OR 4.7, p=0.005), age 75 and above (OR 5.7, p=0.027), osteoarthritis (OR 3.3, p=0.055) and polypharmacy (OR 7.7, p=0.012). Sarcopenia occurs in 26% with female gender (OR 3.1, p=0.029) and SARC-F ≥4 (OR 4.9, p=0.002) as associated factors. Cognitive impairment was present in 20% of participants and associated with illiteracy (p=0.008).

**Conclusion:**

our study shows a high prevalence of geriatric syndromes in older adults in an urban area. Geriatric principles and frailty awareness should be considered in clinical care of older adults in our setting.

## Introduction

Geriatric syndromes (GS) are clinical conditions that are highly prevalent in the aging population. They are not necessarily attributed to a specific isolated underlying disease but rather multifactorial, ultimately leading to substantial vulnerability and reduced quality of life [1,2]. GS include cognitive impairment, delirium, functional decline, falls, urinary incontinence [2-4]. These conditions are associated with recurrent hospitalizations and mortality, as well as significant healthcare expenditure [1,2].

Diseases in the elderly are a major public health problem globally, especially in the high-income countries where the life expectancy is highest. Although they have long been neglected in low-income countries especially in sub-Saharan Africa, geriatric conditions are gaining more attention as the elderly population grows in these countries [5,6]. Indeed, the burden of geriatric diseases is rising in sub-Saharan Africa, with more elderly requiring medical attention, frequent hospitalizations with longer stay and unfortunately high mortality rates [6-8]. Therefore, healthcare systems in low-income countries need some policy shifts to cope with this growing burden of disease in the elderly population. Disability is usually defined as a difficulty in performing activities necessary for independent living [9]. According to a worldwide report, approximately 1 billion people are living with one or more disabling conditions [10,11]. Difficulty in performing basic activities of daily living is present in more than 45% of older adults and influenced by many factors such as older age, cognitive disorders, chronic diseases, limb dysfunction, pain, polypharmacy and high or low body mass index (BMI) [9-13]. Cognitive impairment and dementia are increasing in developing world [14,15]. Cognitive impairment is defined by a progressive decline in some cognitive functions without satisfying diagnosis criteria of dementia. Few studies to determine its prevalence have been conducted in sub-Saharan Africa. The prevalence ranged from 6 to 25% and major risk factors include older age, female gender, cardiovascular diseases and illiteracy [16-18]. Sarcopenia is a muscle disease, with low muscle strength being the principal determinant. Muscle strength is actually the most reliable measure of muscle function. Low grip strength is a predictor of outcomes such as increased functional limitations, poor quality of life as well as longer hospital stay and death [19-21].

Cameroon, a country in Central Africa region reported an increase in life expectancy by 10 years between 1950 and 2015. The healthcare demand in this aging population is increasing, but health system is ill-prepared to meet their needs. The older population was estimated at 1.2 millions individuals in 2018 [22]. As we are writing this paper, there is only one geriatrics-dedicated unit and less than three geriatricians in the whole country [6]. Furthermore, there is a dearth of data on the patterns and determinants of GS in this population. Hence, this study aimed to determine the prevalence of disability, cognitive impairment, sarcopenia and their correlates in an urban population in Cameroon. Such data would inform policies to improve geriatric care in Cameroon.

## Methods

**Study setting, design and participants:** this cross-sectional study was carried out in September 2019, in the Geriatric Unit of the Yaoundé Central Hospital, a 650 bedded hospital located in the capital of Cameroon. This hospital has the lone functional geriatrics-dedicated unit of the country with about 200 hospitalizations per year. Participants were recruited during a health promotion and screening campaign. We included people aged 55 years and above, who provided an informed written consent. This cut-off age to define elderly population and which is lower than that of high-income countries, is a reflection of the country´s life expectancy. We did not include participants who were seriously ill or unable to communicate.

**Data collection:** a pre-designed questionnaire was developed by our team to collect data. The demographic data included age, gender, marital status, current professional activity and educational level (illiterate, primary, secondary, university). Clinical data included past medical history, falls history, number of drugs and comorbidities such as hypertension, diabetes mellitus, cerebrovascular diseases, dementia, cancer, heart disease, osteoarthritis, human immunodeficiency virus (HIV), hepatitis B and C infections. For the purpose of this study, polypharmacy was defined as taking more than four different drugs for chronic diseases. Fall was defined as an unexpected event in which a person come to rest on the floor, the ground or lower level. Falls were assessed by self-report to the question «have you fallen in the past 12 months?»

**Measurements:** geriatric syndromes explored were: disability, sarcopenia and cognitive impairment. Disability was assessed using the Katz Index of independence in Activities of Daily Living (ADLs) [23] and Instrumental Activities of Daily Living (IADLs) adopted from the Lawton scale [24]. The Katz Index of independence in ADL is widely used to assess the older patient ability to perform basic activities of daily living by measuring six functions: bathing, dressing, toileting, transferring, continence and feeding. The Katz Index is sensitive to decline in functional status in various care setting. The Lawton IADL scale is appropriate to assess skills that are more complex than the basic activities of daily living. There are eight functions measured with the scale: ability to use phone, shopping, food preparation, housekeeping, laundry, mode of transportation, responsibility for own medications and ability to handle finances. The Lawton IADL scale has an inter-rater reliability of 0.85 and its correlation with other scales was significant [24]. For the purpose of our study and to limit gender bias, food preparation has been excluded for men. Participants who had difficulty in performing any one of the basic or instrumental activities were classified as living with disability.

Sarcopenia was assessed with the SARC-F questionnaire and the measurement of the muscle strength. SARC-F is an acronym for strength, assistance with walking, rise from a chair, climb stairs and falls. The SARC-F is a screening tool that was evaluated in three large populations, with a high specificity for identifying people at risk of sarcopenia. It consists in a 5 items questionnaire to evaluate patient´s limitations in strength, walking ability, rising from a chair, stair climbing and experiences with falls. Risk of sarcopenia is present if SARC-F ≥4 [25,26]. The measure of the muscle strength was performed by measuring the handgrip strength of the dominant hand with a Jamar dynamometer, sitting upright in a chair. Only one measure was recorded for each participant. According to the European Working Group on Sarcopenia in Older People (EWSGOP), muscle strength is the primary parameter of sarcopenia. Grip strength correlates with strength in other body compartments. The Jamar dynamometer is validated for measuring grip strength [19]. Sarcopenia was present if muscle strength was <30kg for men and <20kg for women.

Cognitive impairment was assessed with the Mini Mental State Examination (MMSE) [27]. The test was administered by a geriatrician or a neurologist. The MMSE is the most widely used cognitive impairment and dementia screening test, including low-literacy settings [28,29]. Its performance in a recent review showed a sensitivity of 0.83 and a specificity of 0.82 in low and middle-income countries [30]. The cut-off values used for the purposes of our study was: 22 for illiterate or primary level, 24 for secondary level and 26 for university level. Numerous studies used different cut-off points for participants with and without formal education to improve the sensitivity and specificity of the test [31-33]. Cognitive impairment was present if the MMSE score was below the cut-off values for each educational level.

**Data analysis:** data were coded, entered and analyzed with the Statistical Package for Social Sciences (SPSS 23.0) for Windows (SPSS, Chicago, Illinois, USA). Quantitative variables were described using mean and standard deviation or median with interquartile range (IQR). Categorical variables were presented with frequencies and percentages. Association between categorical variables were explored using Khi-square test and Fisher´s test. To explore factors associated with geriatric syndromes, we performed univariate and multivariate analysis with odds ratios (OR) and 95% confidential intervals (95% CI). To account for potential confounders, we included in the multivariate model all variables with a p-value <0.2 in the univariate analysis. A p-value of <0.05 was considered statistically significant.

**Ethics approval:** this study has been approved by the institutional board of the Yaoundé Central Hospital (Number 02/ACE/DR/CIE/MINSANTE/ SG/DHCY). An informed consent was obtained from all participants included in this study.

## Results

**Characteristics of participants:** overall, one hundred and four participants were recruited for the study of whom 52.9% (n=55) were female. The median age was 65 (IQR: 62.2 - 70.8) with 47.1% (n=49) of participants aged between 55-64 years. About 60.6% of participants (n=60) presented at least one chronic medical condition, the commonest were hypertension (35.6%, n=37), osteoarthritis (24%, n=25) and diabetes mellitus (8.7%, n=9). Table 1 shows characteristics of all participants.

**Table 1:** characteristics of participants

Characteristics	Women	Men	Overall	p-value
n (%)	55 (52.9)	49 (47.1)	104 (100)	
**Age groups**				
55 - 64	30 (54.6)	19 (38.8)	49 (47.1)	0.108
65 - 74	18 (32.7)	24 (49.0)	42 (40.4)	0.092
75+	7 (12.7)	6 (12.2)	13 (12.5)	0.941
Mean age	66.2 ± 9.98	67.6 ± 6.09	66.8 ± 8.37	0.405
**Marital status**				
In relationship	20 (36.4)	44 (89.8)	64 (61.5)	
Single	35 (63.6)	5 (10.2)	40 (38.5)	0.000
**Educational level**				
Illiterate	8 (14.5)	2 (4.1)	10 (9.6)	0.098
Primary	20 (36.4)	8 (16.3)	28 (26.9)	0.027
Secondary	24 (43.6)	29 (59.2)	53 (51.0)	0.122
University	3 (5.5)	10 (20.4)	13 (12.5)	0.035
Professional activity	12 (21.8)	11 (22.5)	23 (22.1)	1.000
**BMI ranges (kg/m^2^)**				
<20	6 (10.9)	3 (6.1)	9 (8.7)	
20 - 24.9	10 (18.1)	15 (30.6)	25 (24.0)	0.337
25 - 29.9	21 (38.2)	20 (40.8)	41 (39.4)	
30+	18 (32.7)	11 (22.5)	29 (27.9)	
Presence of comorbidities	35 (63.3)	28 (57.1)	63 (60.6)	0.499
Hypertension	21 (38.1)	16 (32.7)	37 (35.6)	
Osteoarthritis	13 (23.6)	12 (24.5)	25 (24.0)	
Diabetes	4 (7.3)	5 (10.2)	9 (8.7)	
Geriatric syndromes (yes)	32 (58.1)	38 (77.6)	70 (67.3)	0.036

BMI: body mass index

**Geriatric syndromes and associated factors:** the prevalence of geriatric syndromes (GS) in the overall population was 67.3% (n=70). Younger age (under 64) seems to protect against the occurrence of GS in univariate analysis (OR 0.3, p=0.012). GS occur significantly in male participants (OR 1.7, p=0.036), in those with BMI <20kg/m^2^ (p=0.029) and in presence of comorbidities (OR 2.3, p=0.049), especially osteoarthritis (OR 4.7, p=0.011). On multivariate analysis, male gender (OR 2.7, p=0.035) and osteoarthritis OR 4.3, p=0.034) were associated with the presence of any GS (Table 2).

**Table 2:** factors associated with the presence of any geriatric syndromes using univariate then multivariate analysis

Variable	uOR (95% CI)	p value	aOR (95% CI)	p value
**Gender**				
Female				
Male	1.7 (1.01 - 2.8)	0.036	2.7 (1.1 - 6.9)	0.035
**Age groups**				
55 - 64	0.3 (0.1 - 0.8)	0.012		0.255
65 - 74	2.02 (0.8 - 4.9)	0.112		0.979
75+	2.9 (0.6 - 14.3)	0.213		
**Marital status**				
In relationship	1.4 (0.6 - 3.3)	0.409		
Single				
**Educational level**				
Illiterate	2.1 (0.4 - 10.3)	0.492		
Primary	0.9 (0.4 - 2.6)	0.942		
Secondary	0.7 (0.3 - 1.7)	0.484		
University	1.1 (0.3 - 3.9)	1.000		
Professional activity	0.7 (0.3 - 1.8)	0.456		
**BMI ranges (kg/m^2^)**				
<20		0.029		1
20 - 24.9	1.3 (0.5 - 3.6)	0.566		
25 - 29.9	0.8 (0.3 - 1.7)	0.495		
30+	0.6 (0.2 - 1.4)	0.240		
Presence of comorbidities	2.3 (0.9 - 5.3)	0.049		0.436
Hypertension	1.2 (0.5 - 2.9)	0.632		
Osteoarthritis	4.7 (1.3 - 17.2)	0.011	4.3 (1.1 - 16.5)	0.034
Diabetes	0.9 (0.2 - 4.1)	0.966		
Polypharmacy	2.4 (0.6 - 8.9)	0.196		0.231

uOR: unadjusted odds ratio, aOR: adjusted odds ratio, 95% CI: 95% confidential interval, BMI: body mass index

Our results showed that 9.6% (n=10) of participants had at least one difficulty with ADLs. Concerning IADLs, difficulty was reported by 37.5% (n=39) of participants with a significant male predominance (p=0.022). In ADLs, the commonest limitations were transferring (80%) and continence (20%). Housekeeping, laundry and shopping caused difficulties in IADLs for the majority of participants. As presented in Table 3, disability in IADLs occurs significantly in male participants (OR 1.6, p=0.022), in those aged 75 and above (OR 7.1, p=0.004), in presence of osteoarthritis (OR 3.4, p=0.008) and polypharmacy (OR 6.8, p=0.001). Those factors remain associated with occurrence of IADL disability in multivariate analysis as shown in Table 3.

**Table 3:** factors associated with disability in IADL using univariate then multivariate analysis

Variable	uOR (95% CI)	p value	aOR (95% CI)	p value
**Gender**				
Female				
Male	1.6 (1.1 - 2.4)	0.022	4.7 (1.6 - 13.7)	0.005
**Age groups**				
55 - 64	0.4 (0.2 - 0.9)	0.029		0.876
65 - 74	1.04 (0.5 - 2.3)	0.918		
75+	7.1 (1.8 - 27.9)	0.002	5.7 (1.2 - 26.2)	0.027
**Marital status**				
In relationship	1	0.999		
Single				
**Educational level**				
Illiterate	1.8 (0.5 - 6.5)	0.496		
Primary	0.6 (0.2 - 1.5)	0.254		
Secondary	1	0.960		
University	1.5 (0.5 - 4.9)	0.548		
Professional activity	0.4 (0.1 - 1.1)	0.077	0.4 (0.1 - 1.5)	0.162
**BMI ranges (kg/m^2^)**				
<20	2.2 (0.6 - 8.9)	0.290		
20 - 24.9	2.5 (1.1 - 6.9)	0.028		0.428
25 - 29.9	0.4 (0.2 - 0.9)	0.026	0.3 (0.1 - 0.8)	0.023
30+	0.8 (0.3 - 2.04)	0.693		
Presence of comorbidities	2.2 (0.9 - 5.1)	0.07		0.523
Hypertension	2.1 (0.9 - 4.7)	0.081	2.9 (0.9 - 9.9)	0.081
Osteoarthritis	3.4 (1.4 - 8.7)	0.008	3.3 (1.1 - 10.9)	0.055
Diabetes	3.8 (0.9 - 16)	0.077		
Polypharmacy	6.8 (2 - 22.9)	0.001	7.7 (1.6 - 38)	0.012

uOR: unadjusted odds ratio, aOR: adjusted odds ratio, 95% CI: 95% confidential interval, BMI: body mass index

About 23.1% (n=21) of participants were at risk of sarcopenia. Sarcopenia was present in 26.2% (n=27) of participants and the median muscle strength was 27 kg (IQR 21.1 - 34.4). Table 4 shows factors associated with sarcopenia. Female gender (OR 3.5, p=0.009), falls history (OR 8.4, p=0.005), SARC-F ≥4 (OR 3.3, p=0.000) and BMI<20kg/m^2^ (OR 4.1, p=0.036) were significantly associated in a univariate model. On multivariate analysis, only female gender (OR 3.1, p=0.036) and SARC-F ≥4 (OR 4.9, p=0.027) were independently associated with the presence of sarcopenia. Cognitive impairment was present in 20.2% (n=21) of participants. The median score was 26 (IQR 24-28). Only educational level was significantly associated with cognitive impairment (p=0.043).

**Table 4:** factors associated with sarcopenia using univariate then multivariate analysis

Variable	uOR (95% CI)	p value	aOR (95% CI)	p value
**Gender**				
Female	3.5 (1.3 - 9.3)	0.009	3.1 (1.1 - 8.6)	0.029
Male				
**Age groups**				
55 - 64	0.5 (0.2 - 2.9)	0.128		
65 - 74	1.2 (0.5 - 2.9)	0.652		
75+	2.8 (0.9 - 9.3)	0.097		
**Marital status**				
In relationship	0.2 (0.1 - 0.6)	0.002		
Single				
**Educational level**				
Illiterate	3.2 (0.9 - 12.1)	0.123	3.03 (0.6 - 15.8)	0.188
Primary	2.04 (0.8 - 5.3)	0.137	2.6 (0.8 - 8.7)	0.108
Secondary	0.6 (0.2 - 1.4)	0.195		
University	0.2 (0.02 - 1.7)	0.176		
Professional activity	1.3 (0.5 - 3.6)	0.601		
**BMI ranges (kg/m^2^)**				
<20	4.1 (1.1 - 16.6)	0.036		
20 - 24.9	0.7 (0.2 - 2.1)	0.494		
25 - 29.9	0.3 (0.1 - 0.9)	0.030	0.3 (0.1 - 1.02)	0.055
30+	2.2 (0.9 - 5.6)	0.091		
Presence of comorbidities	1.1 (0.4 - 2.7)	0.823		
Hypertension	0.9 (0.3 - 2.2)	0.744		
Osteoarthritis	1.5 (0.6 - 3.9)	0.450		
Diabetes	0.8 (0.2 - 4.1)	1		
SARC-F ≥4	3.3 (1.7 - 6.5)	0.000	4.9 (1.6 - 13.7)	0.002
Falls	8.4 (1.5 - 46.4)	0.005	5.5 (0.7 - 43.2)	0.109

uOR: unadjusted odds ratio, aOR: adjusted odds ratio, 95% CI: 95% confidential interval, BMI: body mass index

## Discussion

We found a high prevalence of disability in our study. Our results are similar to those found in Europe, where the disability rate among older people varies between 11 and 44% for ADLs and between 8 and 40% for IADLs [34,35]. In our study, the prevalence was higher in population aged 75 and above, reaching 77% for those reporting limitations in IADLs. In Poland, the odds of having difficulties increased by 8% and 10% with increasing age for ADLs and IADLs respectively [35,36]. The risk of functional disability was four-fold increased in the 80 and above age group in an Irish study [13]. Functional impairment in older people is becoming a major issue in our setting probably because the awareness is still very low among healthcare givers and family members. Factors independently associated with disability in our study were male gender, age ≥75, osteoarthritis and polypharmacy. Similar findings were seen in other studies [37,38]. Housekeeping, laundry and shopping are rarely done by men in our setting, especially when they are in relationship. This can be a confounder in male participants IADLs assessment. Osteoarthritis can impair mobility of older people and causes limitations to perform basic and complex activities. Moreover, limitations due to osteoarthritis can be associated with pain. Some authors demonstrate that the occurrence of disability increases with the number of chronic medical conditions [36,39,40]. Association between disability and polypharmacy is not well described but we can assume polypharmacy is usually associated to multimorbidity.

Sarcopenia was highly prevalent in our study population. This finding is in concordance with previous studies [41,42]. But our prevalence is higher than in other studies conducted in Nigeria and Brazil [43,44]. Because of the variability of the instruments used to screen sarcopenia, the cut-off values, age of the study population and comorbidities, the prevalence of sarcopenia varies widely [19,45,46]. According to the European Working Group on Sarcopenia in Older People (EWGSOP), handgrip strength of the dominant hand is the best evidence at date, to assess general muscle strength in a clinical setting or in community health care [19]. Older Africans have been found to have higher muscle mass than their Caucasians counterparts. However, new research showed that muscle strength could be addressed independently of muscle mass [47,48]. In our study, women were at a higher risk of having sarcopenia. Women undergo an accelerated loss of muscle mass at an earlier age than men beginning at the time of menopause. Our results are in line with other studies [43,49,50]. We reported a higher risk of sarcopenia among individuals with history of falls. The consequences of falls can be disastrous since they are associated with physical disability, functional impairment and increased morbidity and mortality [51-53].

Cognitive impairment was present in 20.2% of participants in this study and was associated with illiteracy. This prevalence is lower than the 33.3% obtained in rural Cameroon [54]. However, the prevalence of cognitive impairment varies widely in Cameroon and in other African countries [16,55,56]. Discrepancies found between different studies can be explained by the multiplicity of tools and cut-off values. Indeed to improve the sensitivity of the MMSE in our population, it was important to adjust the cut-offs to educational level [31-33]. However, we were not able to determine related factors with statistical significance, this could be explained by our small sample size.

The burden of geriatric conditions is increasing in sub-Saharan Africa and needs to be identified to plan preventive and therapeutic approach. According to World Health Organization, 2020-2030 is the decade of healthy aging. It is therefore important to raise awareness of healthcare givers about health issues of the older adults. Further studies are highly needed in this rare area to provide concrete actions for a healthy aging in our setting.

**Limitations:** although we expect this study to help increasing public health awareness concerning geriatric conditions, it should be interpreted considering some limitations. Our data might not reflect the entire elderly population in our setting as we conducted a hospital-based study in one center. Another limitation was the fact that factors associated with cognitive impairment such as mood disorders were not taken into account.

## Conclusion

This study shows evidence for high prevalence of GS in Cameroonian older people in an urban setting. The burden of GS needs to be identified early to minimize the related complications. Further research among community-dwelling and hospitalized older individuals are needed to motivate the government to put in place policies and implement appropriate preventive measures.

### What is known about this topic

- Geriatric syndromes are associated with substantial disability, poor quality of life and mortality in the elderly;
- Assessment of functional status, cognition and frailty is important to prevent adverse events in older patients.

### What this study adds

- To our knowledge, this study is the first in sub-Saharan Africa to assess geriatric conditions in outpatients including disability and sarcopenia;
- This study shows evidence of high prevalence of geriatric syndromes in outpatients and can help to initiate a community-dwelling screening of those conditions in older adults in our setting.
